# Established and candidate transthyretin amyloidosis variants identified in the Saudi population by data mining

**DOI:** 10.1186/s40246-021-00351-2

**Published:** 2021-08-11

**Authors:** Mohamed Abouelhoda, Dania Mohty, Islam Alayary, Brian F. Meyer, Stefan T. Arold, Bahaa M. Fadel, Dorota Monies

**Affiliations:** 1 Department of Genetics, King Faisal Specialist Hospital and Research Centre, P.O. Box 3354, Riyadh, 11211 Saudi Arabia; 2 Heart Center, King Faisal Specialist Hospital & Research Center, Riyadh, Saudi Arabia; 3 Al-Faisal University, College of Medicine, Affiliate Harvard Medical School International, Riyadh, Saudi Arabia; 4 Pfizer Inc., Jeddah, Saudi Arabia; 5 Computational Bioscience Research Center, Division of Biological and Environmental Sciences and Engineering, King Abdullah University of Science and Technology, Thuwal, Saudi Arabia

**Keywords:** Transthyretin, Amyloidosis, Familial, Saudi population, Epidemiology

## Abstract

*****Background***:**

Familial transthyretin (TTR) amyloidosis (ATTR) is an autosomal dominant disease with significant phenotypic heterogeneity. Its prevalence in Saudi Arabia has not previously been investigated. An existing exome variant database of Saudi individuals, sequenced to globally investigate rare diseases in the population, was mined for *TTR* variants and filtered for missense mutations resulting in single amino acid changes. A total of 13,906 Saudi exomes from unrelated individuals were analyzed blindly.

*****Results***:**

Three *TTR* variants known to be associated with ATTR amyloidosis were identified. Additionally, three novel *TTR* mutations were identified. Structural analysis of the three novel variants suggests that at least two could be amyloidogenic. The most common variant associated with amyloidosis was p.Val142Ile (allele frequency 0.001). Further investigation of these variants and their translation to clinical practice may help to diagnose, monitor, and manage patients with ATTR amyloidosis.

*****Conclusion***:**

Multiple *TTR* variants potentially associated with systemic ATTR amyloidosis were identified in the Saudi population. Early diagnosis and intervention, facilitated by familial genetic testing of patients with ATTR amyloidosis, may benefit in the management of this disease. Early diagnosis could be enhanced through inclusion of ATTR variants in existing population-based screening programs.

**Supplementary Information:**

The online version contains supplementary material available at 10.1186/s40246-021-00351-2.

## Background

The tetrameric transthyretin (TTR) protein is stabilized by the binding of thyroid hormone or retinol-binding protein 4 (RBP4), preventing its dissociation into monomers that can aggregate into amyloid fibrils [1–3]. Pathogenic accumulation of amyloid TTR (ATTR) can be promoted either by misfolding of the wild-type protein, as seen in wild-type systemic amyloidosis, or by the presence of inherited or de novo mutations in the *TTR* gene on Hsa18 that result in single amino acid changes to the protein [4]. These changes may destabilize the tetramer by impeding binding to stabilizing thyroxine or RBP4. More than 120 hereditary *TTR* mutations that increase the rate of formation of amyloid fibrils have been identified [5, 6]. Hereditary ATTR is an autosomal dominant disorder in which allelic heterogeneity impacts both penetrance and clinical manifestations [7]. Some variants are mostly associated with polyneuropathy, some can induce either early or late onset cardiomyopathy (CM), while for others, mixed phenotype (neuro and cardiological) may be present [7]. Phenotypes arising from the same variant can also differ between individuals due to several factors such as age, gender ethnicity, and other unknown factors [8]. Polyneuropathy resulting from ATTR is progressive and involves autonomic and sensorimotor neural dysfunction that results in significant disability and death. Life expectancy of patients with hereditary ATTR ranges from 7 to 11 years after the diagnosis is established [9]. CM associated with ATTR (ATTR-CM) tends to have a worse prognosis than polyneuropathy, with a life expectancy of 2 to 6 years following diagnosis [7].

Given the progressive nature of ATTR and the poor prognosis for patients with ATTR-CM and ATTR-polyneuropathy, early diagnosis is crucial to permit timely management. In clinical practice, a timely diagnosis remains difficult to achieve: symptomatic patients with amyloidosis experience an average delay of 4 years before a correct diagnosis is established [10, 11]. A recent review recommended genetic screening of at-risk family members of patients with ATTR amyloidosis to identify pre-symptomatic individuals with pathogenic *TTR* variants, in order to allow early diagnosis and possibly early therapeutic intervention [12]. However, such an approach requires a good understanding of the prevalence of hereditary ATTR and of the clinical manifestations of *TTR* variants in the local population. At present, Arab populations are underrepresented in ATTR databases, thus limiting our ability to adopt such an approach in Saudi Arabia. Large-scale national genomic data mining can be very useful to establish frequency of pathological genetic variation resulting in rare diseases such as amyloidosis [13, 14]. This type of study also offers the opportunity to identify novel variants that are potentially unique to the studied population and may have important epidemiologic, diagnostic and potentially therapeutic implications.

The objectives of this study are (1) to analyze a national exome database in order to identify the incidence of known and candidate pathogenic variants associated with ATTR in Saudi Arabia, and (2) to investigate the amyloidogenic potential of novel candidate *TTR* variants.

## Results

Analysis of the 13,906 Saudi exomes identified 158 *TTR* variants. Of these, 28 were in coding or flanking regions. These were further reduced by removal of synonymous, nonsense and splice variants leaving 12 potentially amyloidogenic variants that were further investigated (Table 1 Two mutations are known to negatively impact function of the TTR protein, resulting in amyloidosis: c.238A>G(p.Thr80Ala) and c.424G>A(p.Val142Ile) [5, 14–16]. The most frequent was c.424G>A(p.Val142Ile) (0.001), whereas c.238A>G(p.Thr80Ala) (0.00004) was less frequent (Table 1). Variant c.239C>T(p.Thr80Ile) affects a hotspot for pathogenic mutations (same amino acid) and according to ACMG guidelines and Varsome should be considered as a pathogenic mutation [17, 18]. Three variantsc.368G>A(p.Arg123His), c.370C>T (p.Arg124Cys), and c.385G>A (p.Ala129Thr) were classified as variants of uncertain significance (VUS) having no known effect on TTR function [14, 17]. Three further variants were classified as benign/likely benign (Table 1). Three novel *TTR* variants were identified by this study: c.404C>T (p.Ser135Phe), c.428C>T (p.Thr143Ile), and c.298A>G (p.Lys100Glu) and were among the rarest in this cohort, with only 1 allele of each being observed (Table 1).

**Table 1:** Identified missense *TTR* variants

***TTR*** variant	References	Number of heterozygous	Number of homozygous	Allele frequency
Known to affect TTR function				
c.239C>T:p.Thr80Ile	[17]	2	0	0.00007191
c.238A>G:p.Thr80Ala	[5, 16, 19]	1	0	0.00003596
c.424G>A:p.Val142Ile	[5, 16, 19]	28	0	0.00100676
Variants of unknown significance				
c.368G>A:p.Arg123His	[17]	2	0	0.00007191
c.370C>T:p.Arg124Cys	[17]	6	0	0.00021573
c.385G>A:p.Ala129Thr	[17]	3	0	0.00010787
Benign/likely benign				
c.76G>A:p.Gly26Ser	[17]	178	0	0.00640012
c.140A>G:p.Asn47Ser	[17]	2	0	0.00007191
c.328C>A:p.His110Asn	[17]	239	6	0.00902488
Novel variants				
c.404C>T:p.Ser135Phe	[17]	1	0	0.00003596
c.428C>T:p.Thr143Ile	[17]	1	0	0.00003596
c.298A>G:p.Lys100Glu	[17]	1	0	0.00003596

**Fig. 1:**
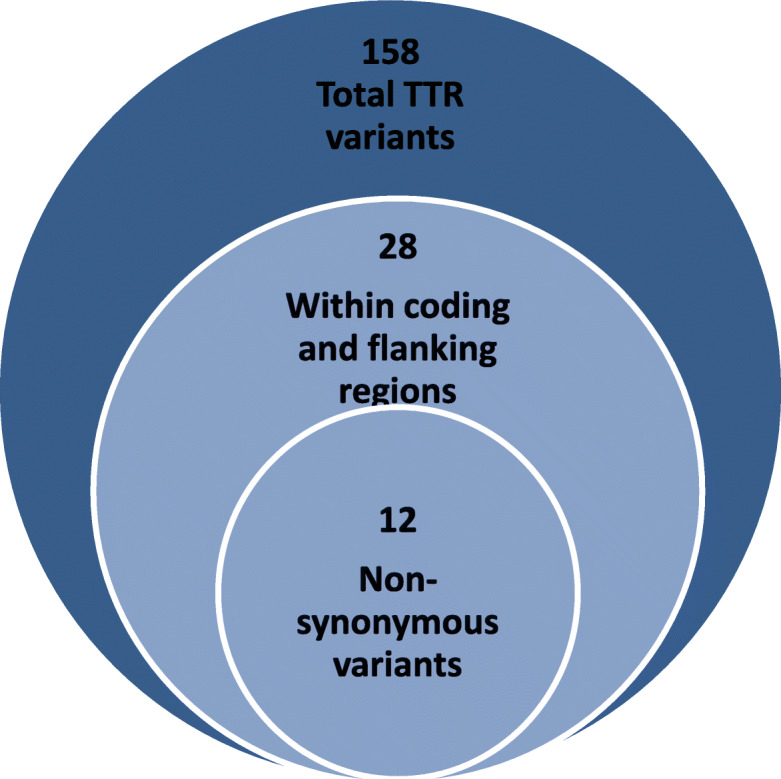
Computational structural analysis of novel variants. Mapping of the mutations onto the TTR tetramer. **A** The TTR tetramer (each chain is color-coded) bound to RBP (gray) and thyroxin (thy, red). The mutated residues are shown as sphere models, corresponding to the frames with the zoomed-in regions. For clarity, not all residues are labeled. Note that the RBP binding environment of the same residue is different in different TRR chains. **B**–**D** The local environment of each mutated residue. The side chains of the variants are shown in green. **C** The red spheres illustrate the steric clashes resulting from the mutation

Several high-resolution crystallographic structures exist for the TTR tetramer, in its apo-form, bound to stabilizing compounds, and bound to RBP4. One TTR tetramer binds to two RBP4 (Fig. 1 A). One novel variant, c.298A>G (p.Lys100Glu), is located at the interface between two TTR molecules and RBP4 (Fig. 1 B). Its replacement with an oppositely charged glutamic acid will alter the charge balance of this area, thus affecting association with RBP4. A second, c.404C>T (p.Ser135Phe) novel variant sits at the interface between the TTR tetramer and thyroxine. Replacing Ser135 with a much larger phenylalanine introduces steric interference and leads to substantial changes in the thyroxine binding pocket because of the proximity of the four Ser135 from the four TTR tetramer chains (Figure 1 C). c.428C>T (p.Thr143Ile) is located at the C-terminus of TTR. Its replacement with an isoleucine does not create steric hindrance and does not impact intra or intermolecular interactions. If a strong association of this variant with phenotype is established, then it may be related to other mechanisms, such as folding dynamics (Fig. 1 D).

## Discussion

This is the first study to identify and investigate the prevalence of pathogenic *TTR* variants in the population of Saudi Arabia, a population that is not represented in current databases. The investigation of *TTR* variants in a previously unstudied population would be expected to provide insights into the prevalence of known amyloidogenic variants and potentially identify novel variants that might be associated with systemic amyloidosis. Of the variants known to be associated with amyloidosis, c.424G>A(p.Val142Ile) was the most frequently identified in the Saudi population with an allele frequency of 0.001, similar to the frequency reported in a multinational database [14]. This variant is most often detected in individuals of African descent, with approximately 3% of African-Americans carrying at least one copy of the gene (allele frequency 0.0173) [20]. It is known to be associated with late onset CM, more commonly in men (Table 2) [8, 20–23]. Another known amyloidogenic variant, c.238A>G(p.Thr80Ala), was found at a frequency of 0.00004 in our database, suggesting that it is rare in the Saudi population. This variant is relatively common in Ireland and the UK with 1.1% of the northwest Irish population being carriers [15, 16]. It has also been found in other regions associated with Irish immigration, as well as in populations with no known Irish or UK ancestry [8]. This variant is associated with cardiac amyloidosis as well as autonomic and peripheral neuropathy (Table 2) [24, 25].

**Table 2:** Phenotypes of known amyloidogenic *TTR* variants with alleles identified in the Saudi population

Variant	Phenotype
c.424G>A(p.Val142Ile) [8, 13, 21–23]	• Late-onset cardiomyopathy (over the age of 65), more commonly in men • Low amyloid load, remaining subclinical in many carriers
c.238A>G(p.Thr80Ala) [8, 15, 16, 24, 25]	• Age of onset in seventh decade of life • Cardiac amyloidosis and autonomic and peripheral neuropathy • Gastrointestinal disorders are common. Carpal tunnel syndrome precedes other symptoms in nearly three quarters of patients
c.239C>T(p.Thr80Ile) [5, 17]	• Qualifies as a dense hot-spot and pathogenic mutation by Varsome, but there are no reports of the clinical manifestation • It is similar to p.Thr80Ala, as both substitute a native uncharged polar residue with a non-polar residue

Our study also identified two individuals with another known amyloidogenic variant, c.239C>T(p.Thr80Ile), with an allele frequency of 0.00007. There are few published reports of this variant, suggesting that it is rare. The phenotype associated with this variant has not been previously reported in the literature. It is absent in major databases (1000 Genomes Project, Exome Variant Server, Genome Aggregation Database), consistent with it being ethnically restricted. However, it is recorded in the Mutations in Hereditary Amyloidosis database as being amyloidogenic [5] and its similarity to the p.Thr80Ala variant suggests that its clinical implications might be predicted (Table 2). Interestingly, some of the most common variants identified in other populations are not observed in the Saudi database of 13,906 individuals. These include c.148G>A(p.Val50Met), the most common variant in Western Europe [8]; p.Leu131Met, a variant with cardiac manifestations predominantly found in Denmark [8, 26]; p.Ile88Leu, which is seen predominantly in the Italian population [8, 27] and has primarily cardiac manifestations [27]; p.Val50Ala, p.Ala117Ser, and p.Gly103Arg, the most common amyloidogenic variants in the Chinese population [28]; and p.Ser70Arg, the most common variant in Mexico [29]. The absence of these variants from our database does not imply that they are absent in the Saudi population. However, if present, they are likely to have a very rare prevalence. Our findings further indicate that there is significant population variation in the prevalence of *TTR* variants, including some variants that are relatively restricted to certain ethnic groups. The fact that some of the *TTR* variants identified in the Saudi database have not been previously reported suggests that they might be restricted to the Saudi and possibly neighboring populations. Two of the three novel variants in our study were predicted by computational structural analysis to result in a reduced capacity of the TTR protein to associate with thyroid hormone or RBP4. These associations are thought to aid in the stabilization of the TTR tetramer and prevent it from dissociating into fibril-forming monomer aggregates [1–3]. Thus, it is possible that patients carrying these variations are at risk of developing future clinical amyloidosis. However, this in silico analysis may be considered as only “predictive” and further investigations are required to confirm the clinical implications of these novel variants.

The study is limited by sample size (13,906) which, while relatively large, will not identify very rare or *de novo* ATTR alleles within the population. The use of a cohort of 13,906 unrelated individuals, primarily sequenced to investigate rare inherited diseases globally, is not expected to introduce any bias in the ascertainment of allele frequencies. Individuals represented in our database originate from a large geographic area encompassing 5 different regions within Saudi Arabia, thus allowing adequate estimation of *TTR* variants that are not vanishingly rare in the country [30, 31]. Another limitation of our study is that the interpretation of the clinical implication of novel pathogenic variants is restricted, as there is little clinical information and follow-up linked to the exome data. While one may predict that at least two of the three novel variants identified in our database are potentially amyloidogenic using structural analysis, without family histories and segregation, one cannot assume that such variants will ultimately result in clinical ATTR amyloidosis. This limitation also applies to variants of known function. How these data link to clinical manifestations in the population of Saudi Arabia remains unknown and, therefore, no comparison of penetrance or manifestations of identified variants between Saudi and other studied populations can be made. Such information could provide a valuable insight on how genetic background may influence the penetrance and manifestations of *TTR* variants. Furthermore, the prevalence of identified variants cannot be compared to the number of patients with a known diagnosis, precluding any attempts to determine if there is significant underdiagnosis in Saudi Arabia. Studies in other populations have noted the potential for extensive underdiagnosis [32, 33].

The data described in this manuscript suggest that there are *TTR* variants potentially associated with amyloidosis in Saudi Arabia and highlight the need for further clinical data regarding this patient population. Reports from other populations suggest that a concerted effort is required to identify, monitor, and manage individuals with pathogenic *TTR* variants. This approach would likely allow therapeutic intervention before considerable deposition of amyloid fibrils induces symptoms and advanced organ damage [12, 34]. In Saudi Arabia, such efforts could focus on: the routine genetic testing of patients with phenotypes that raise suspicion of amyloidosis irrespective of age; the development of genetic testing programs for relatives of patients with known ATTR amyloidosis and the utilization of existing newborn and pre-marital genetic screening programs to identify carriers of pathogenic *TTR* variants; the development of a consensus on optimal monitoring and management of patients with pathogenic *TTR* variants, including appropriate genetic counseling for family members; and finally, establishing a registry for patients with amyloidosis. In individuals with a phenotype suspicious for amyloidosis such as hypertrophic cardiomyopathy, the presence of a *TTR* mutation has diagnostic implications and points away from a myocardial sarcomeric disease and towards amyloidosis. The identification of potentially pathogenic *TTR* mutations has important clinical implications for the classification, diagnosis, and treatment of amyloidosis. Within the disease context, the detection of a mutation allows one to classify TTR amyloidosis as hereditary rather than wild-type and should prompt consideration for genetic screening of siblings. Genetic screening conducted on siblings of patients with the hereditary form allows for the detection of mutation carriers who are at risk of developing future clinical amyloidosis. Saudi Arabia benefits from extensive existing screening programs, meaning that there is infrastructure already in place to facilitate screening for *TTR* variants in people known to be at risk and in the wider population.

**Fig. 2:**
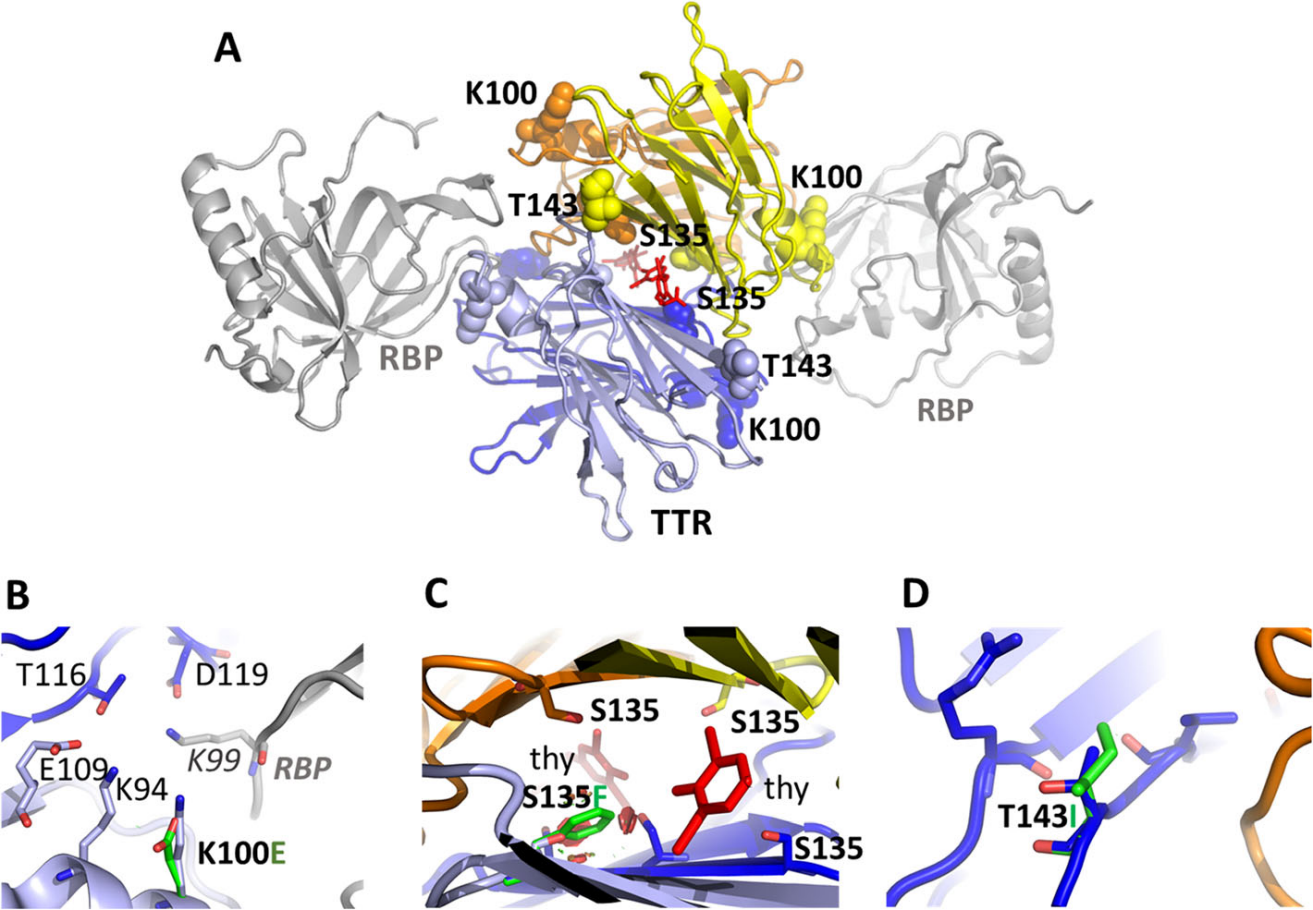
Framework for initial assessment and follow-up of asymptomatic carriers of *TTR* mutations

The aim of these screening programs is to identify patients before they become symptomatic. A recent article by Conceição and colleagues [12] on early diagnosis and follow-up cautioned that it is important to minimize any anxiety that could be caused through over-medicalization from the knowledge of carrier status and repeated follow-up. In order to determine how these patients should be monitored in Saudi Arabia, it is therefore important to carefully consider and gain consensus on when patients should begin to be monitored and at what time intervals; which clinicians and other healthcare professionals should be involved; what should be done for patients with *TTR* variants of currently uncertain effect; when treatment should be initiated; and which tests should be performed to monitor patients. In this context, biomarkers such as serum TTR levels or urine RBP4 [35] have potential for more widespread use in the clinic. Furthermore, current imaging tools can allow the detection of organ involvement at a subclinical stage, before overt morphological abnormalities become evident. For example, cardiac imaging using Tc-labelled phosphate compounds with high affinity for TTR allows early diagnosis of amyloid myocardial deposition, not only at a pre-symptomatic stage, but also before an increase in wall thickness is detected by echocardiography and prior to the development of electrocardiographic abnormalities [36]. A recently published Japanese expert opinion recommended periodic clinical assessment as well as various investigations in monitoring asymptomatic gene mutation carriers [34]. With the advent of drugs that help stabilize the TTR tetramer, reduce tissue deposition and possibly slow disease progression, [37] an early diagnosis guided by genetic screening should be strongly pursued. Such drugs may well prove to be more effective if administered before the development of overt tissue involvement and organ damage. Figure 2 illustrates a framework for the initial assessment and follow-up of individuals who are asymptomatic carriers of *TTR* mutations.

## Conclusion

We present data to suggest that the Saudi population has a unique subset of *TTR* variants and possibly lacks variants commonly present in other patient populations. Additionally, we identified the presence of novel, potentially pathogenic, TTR variants. Our findings support the need for a nationwide registry and a framework for genetic testing with systematic patient evaluation and follow-up.

## Methods

### TTR variant data mining and filtration

**Fig. 3:**
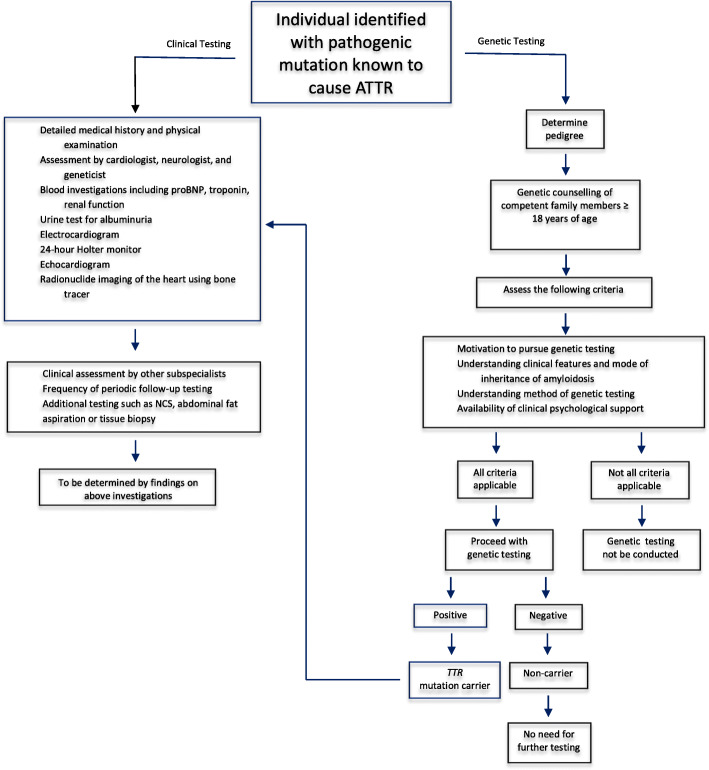
Schematic representation of the filtration process

The database comprises 13,906 consecutive exomes from unrelated Arab individuals, sequenced between 2015 and 2019 as part of a global investigation of rare diseases in the Saudi population [30, 31, 38]. To the best of our knowledge, no sample biases likely to enrich or deplete *TTR* variants are present. The database includes 53% males and 47% females with an average age of 9 years. All *TTR* (NM_000371) variants were selected from the database for analysis and were further filtered to include non-synonymous and potentially pathogenic variants (Fig. 3, Table S2). Synonymous variants and other changes not expected to affect protein folding were removed (Table S1), but may be used as valuable markers in early detection of disease. The remaining variants were classified as pathogenic, likely pathogenic, variants of uncertain significance (VUS), or benign/likely benign according to recommendations published by the American College of Medical Genetics [18] as ascertained by Varsome [17]. We used available databases and published data to identify variants associated with clinical ATTR amyloidosis in other patients. The following publicly available sources were used: ClinVar (www.ncbi.nlm.nih.gov/clinvar/), the Mutations in Hereditary Amyloidosis database (www. amyloidosismutations.com), the Human Gene Mutation database (www.hgmd.cf.ac.uk), and literature from PubMed (www.PubMed.com).

### Computational structural analysis of novel mutations

Novel variants were characterized with computational structural analysis that used thyroxin-bound TTR (PDB ID: 2rox) and RBP-bound TTR (PDB ID: 2wqa) as a reference, to identify and analyze the structural and functional repercussions associated with each variant. The PyMOL program (pymol.org) was used to inspect the models obtained for each variant.

## Supplementary Information

**Additional file 1: Table S1.** List of synonymous variants and other changes not expected to affect TTR protein folding.

**Additional file 2: Table S2.** Each category of *TTR* variants in the filtration process.

## Data Availability

Due to the nature of this research, participants of this study did not agree for their data to be shared publicly, so supporting data is not available.
